# Augmented Feedback in Post-Stroke Gait Rehabilitation Derived from Sensor-Based Gait Reports—A Longitudinal Case Series

**DOI:** 10.3390/s25103109

**Published:** 2025-05-14

**Authors:** Gudrun M. Johansson, Fredrik Öhberg

**Affiliations:** 1 Department of Community Medicine and Rehabilitation, Umeå University, SE-901 87 Umeå, Sweden; gudrun.johansson@umu.se; 2 Department of Diagnostics and Intervention, Biomedical Engineering and Radiation Physics, Umeå University, SE-901 87 Umeå, Sweden

**Keywords:** augmented feedback, gait, kinematics, wearable sensors, stroke

## Abstract

Wearable sensors are increasingly used to provide objective quantification of spatiotemporal and kinematic parameters post-stroke. This study aimed to evaluate the practical value of sensor-based gait reports in delivering augmented feedback and informing the development of home training programmes following a 2-week supervised intensive intervention after stroke. Four patients with chronic stroke were assessed on four occasions (pre- and post-intervention, 3-month, and 6-month follow-ups) using clinical gait tests, during which a portable sensor-based system recorded kinematic data. The meaningfulness of individual changes in gait parameters was interpreted based on established minimal detectable change values (MDC). Three participants improved their gait speed, joint angles, and/or cadence in the Ten-Metre Walk Test, and three participants improved their walking distance in the Six-Minute Walk Test. The improvements were most evident at the 3-month follow-up (with the most obvious changes above MDC estimates) and indicated the reappearance of normal gait patterns, adjustments of gait patterns, or a combination of both. Participants showed interest in and understanding of the information derived from the gait reports (ratings of 5–10 out of 10). In conclusion, augmented feedback derived from gait reports provides a valuable complement to traditional clinical assessments used in stroke rehabilitation to optimize treatment outcomes.

## 1. Introduction

After a stroke, many survivors experience decreased walking capacity, manifested by reduced gait speed and endurance [1]. Walking is a fundamental activity that is necessary for maintaining independence in daily life [2], and gait speed is a strong predictor for return to employment for persons post-stroke [3]. Consequently, regaining walking ability is an important goal in stroke rehabilitation. Recommended interventions to improve walking ability include supervised intensive therapy, repetitive task-specific training, and fitness training [4]. A key component of these interventions is the incorporation of feedback mechanisms, which play a crucial role in motor skill reacquisition after stroke [5].

Task intrinsic feedback is the sensory–perceptual information that is a natural part of performing a skill [6], which is often impaired in individuals after a stroke. Extrinsic feedback, also called augmented feedback, is provided externally by a therapist or device and involves adding to or improving task intrinsic feedback [6]. Augmented feedback can be categorized as knowledge of results (KR) or knowledge of performance (KP) [7]. KR relates to achieving the goal of the performance or the externally presented performance outcome, whereas KP relates to the movement characteristics that lead to the performance outcome [8]. KP can enhance motor learning by offering information about movement quality during or after task completion [9]. In stroke rehabilitation, KP is proposed to have more lasting benefits than KR [5,10].

In clinical practice, observational assessments are commonly used to evaluate movement quality. Standardized gait tests, such as the Ten-Metre Walk Test (10MWT) and the Six-Minute Walk Test (6MWT), are well-established tools for assessing spatiotemporal parameters like speed and endurance in stroke rehabilitation [1,11,12,13]. However, these tests do not capture detailed kinematic information, which is essential for distinguishing between true motor recovery—defined as the reappearance of pre-stroke movement patterns—and compensation, which involves alternative movement strategies [14]. Clinical gait assessments by therapists provide qualitative insights into movement patterns, but they are inherently subjective and may lack consistency. Three-dimensional gait analysis (3D-GA) systems offer objective kinematic data [15,16] but are often impractical for routine clinical use due to their complexity and cost. Wearable inertial measurement units (IMUs) provide a promising alternative, allowing for detailed kinematic gait analysis in clinical settings and during standardized gait tests [17]. Despite the increasing use of IMUs for post-stroke gait analysis, their clinical utility in decision-making and rehabilitation planning remains underexplored [18].

This study assesses the practical value of sensor-based gait reports in delivering augmented feedback and informing the development of home training programs following a supervised intensive intervention. Specifically, this case series (i) explores the influence of augmented feedback derived from sensor-based gait reports on spatiotemporal and kinematic parameters among individuals with chronic stroke, and (ii) describes the participants’ perceptions and experiences of receiving augmented feedback. By integrating objective kinematic analysis with subjective feedback given to the participants, this study provides novel insights into the role of wearable IMU sensors in stroke rehabilitation and their potential to enhance clinical decision-making and rehabilitation outcomes. Additionally, the qualitative feedback given to the participants highlights both the benefits and challenges of implementing augmented feedback in real-world rehabilitation settings.

## 2. Materials and Methods

### 2.1. Case Description

Persons with chronic stroke were recruited from a scheduled supervised intensive therapy programme offered by a clinic at the University Hospital of Umeå. This training utilizes a modified approach of Lower Extremity Constraint-Induced Movement Therapy (LE-CIMT) that is considered feasible in group settings of 3–4 patients with stroke [19,20]. Groups were arranged at the clinic when there were enough suitable and motivated patients, usually one group per semester. All four individuals who signed up for the current programme agreed to participate in this study. Criteria for inclusion were as follows: (1) a diagnosis of first stroke in adulthood, (2) able to understand verbal and written instructions, and (3) able to walk with or without aids. Exclusion criteria included other diseases or injuries that could interfere with the treatment or outcomes of this study. This study was approved by the Regional Ethical Review Board, and the participants received written and verbal information before providing written consent in accordance with the Declaration of Helsinki (Dnr. 2018/236-31, 2022-02935-02). Sensorimotor functioning was assessed at admission according to the Fugl–Meyer Assessment of Lower Extremity (FMA-LE) [21]. All participants had a range of residual motor impairments, and three of them had sensory deficits (Table 1). They could walk without aids (however, Participant D needed a walker for longer distances).

### 2.2. Outcome Measures

The 10MWT and the 6MWT were conducted on four occasions: pre-intervention (i.e., the day before the start of the intervention), post-intervention (i.e., the day after the end of the intervention), 3-month follow-up, and 6-month follow-up. First, the participants were asked to walk 10 m without shoes at a comfortable speed, and then at a fast speed without running. Thereafter, the participants were instructed to walk back and forth along a straight 25-metres path as many times as possible for a duration of 6 min. None of the participants expressed a need for rest during the test.

The 3D-GA data were collected simultaneously during gait tests performed in a corridor. The portable inertial sensor-based system MoLab^TM^ (AnyMo AB, Umeå, Sweden) [22] was used. The system includes inertial measurement units (IMUs) that include three types of sensors: a triaxial accelerometer (16-bit, range ± 16 g), a triaxial gyroscope (16-bit, range ± 2000°/s), and a triaxial magnetometer (13-bit, range ± 1200 μT). In the current study, only the triaxial accelerometer and the triaxial gyroscope were used since the study environment had some magnetic disturbances that could not be handled. The sampling frequency for the sensors was set to 100 Hz to ensure that the captured data were accurate and provided a comprehensive analysis of body motion.

Before starting this study, the triaxial accelerometer in each sensor was dynamically calibrated by performing a slow rotation around all its sensitive axes. The accelerometers’ offset and scaling factors were adjusted to ensure that the length of the accelerometer vector always lies on a unit sphere. To achieve this, each accelerometer was subjected to a slow and controlled rotation around all three of its sensitive axes. This rotation helped capture the full range of accelerometer outputs. During the rotation, acceleration data were continuously recorded. These data were used to identify any offsets and scaling discrepancies in the accelerometer readings. The recorded data were analysed to determine the offset for each axis. These offsets were then subtracted from the raw accelerometer readings to correct for any bias. The scaling factors for each axis were calculated to ensure that the magnitude of the accelerometer vector was equal to 1. This calculation was performed by normalising the accelerometer readings so that they lie on a unit sphere. The triaxial gyroscopes were calibrated at the beginning of each measurement while the participant stood quietly. The baseline offset recorded during this period was then subtracted from all collected gyroscopic data for that measurement. This calibration was performed to minimize drift in the subsequent angle measurements.

**Figure 1:**
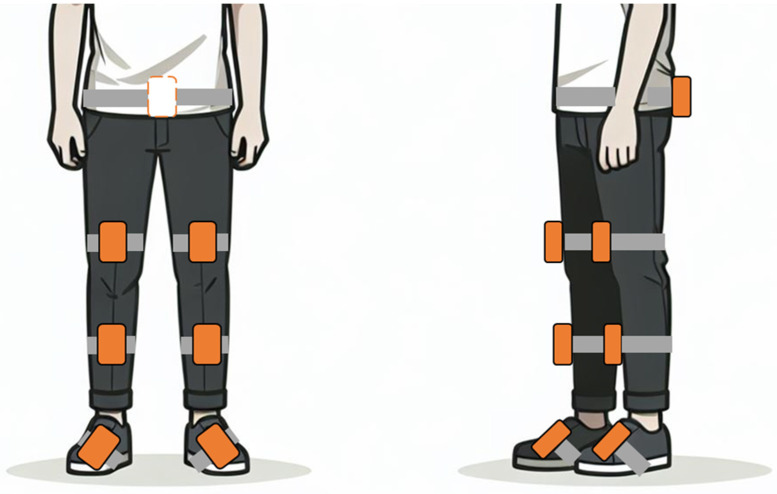
Visualization of a test person in the frontal and sagittal planes with seven movement sensors placed on the pelvis (one sensor), thighs (one on each thigh), shanks (one on each shank), and feet (one on each foot). The sensors capture kinematic data to analyse movement patterns during gait assessment. This study focuses on sagittal plane movements to evaluate gait kinematics.

Seven lightweight sensors were fixed with elastic straps on the pelvis, each thigh, shank, and foot (Figure 1). A comprehensive gait report was compiled, featuring time-normalized gait curves from the 10MWT at each time point. These gait curves were compared to those of a reference group. The reference database contained data from 100 participants (aged 40–75 years old) without gait disturbances, who performed standardized gait assessments collected by the same portable motion analysis system used for the participants.

To investigate the practical application of sensor-based gait reports, participants completed a questionnaire that was specifically designed for this study at the 6-month follow-up (Appendix A). The participants’ perceptions of understanding and interest in sensor-based information were assessed using a 10-point scale (0 = *very bad*, *not at all interested*, or *do not agree at all*, whereas 10 = *very good*, *very interesting* or *completely agree*). In addition, participants had the opportunity to add comments to their scored answers.

### 2.3. Intervention

The participants underwent supervised training focused on the lower limbs for 6 h per day over ten consecutive weekdays. An experienced physiotherapist provided supervision during the intervention. The use of a physical restraint (orthosis) for the non-affected lower limb was limited to 40 min per day and only during supervision due to safety reasons. The daily training was organized into sessions lasting 30–60 min, with each session focusing on strength, balance, coordination, gait, and functional training in different orders [19,23]. The training was performed as a group intervention, but the exercises were individually adapted and gradually increased in degree of difficulty and complexity. The intervention consisted of overground and treadmill gait training, strength training during functional tasks and with strength training equipment, weight-bearing transfers in different directions, balance training on various surfaces including Wii games, ball activities, cycling, and passive muscle stretching. Gait training was also performed in everyday environments, both indoors and outdoors. Also, walking with bent hips and bent knees (crouch gait) was used as a form of forced-use and non-constraint exercise [24]. In addition to the modified LE-CIMT in the clinic, participants were instructed to use the more affected limb in real-life situations and physical activities. They were recommended to perform individual home training programmes several times a week between the post-intervention and the follow-ups.

### 2.4. Augmented Feedback

**Figure 2:**
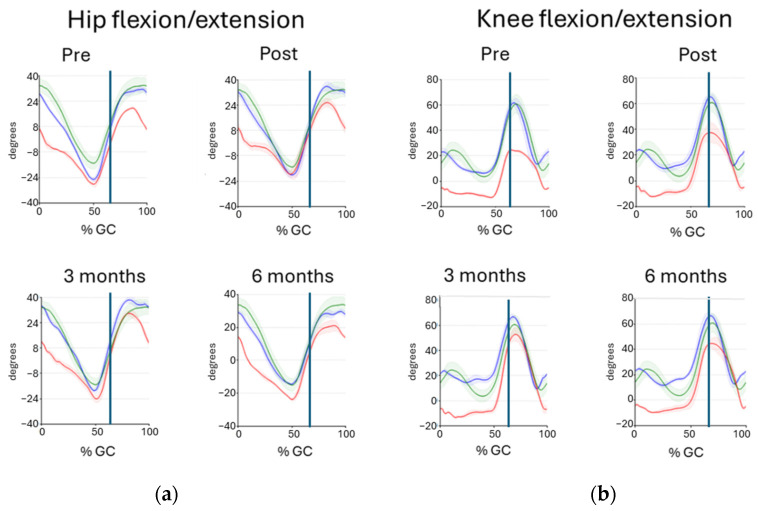
Sagittal joint angles time-normalised to the gait cycle (GC) for the hip (**a**) and knee (**b**) of Participant A during the 10MWT at a comfortable speed. Vertical blue lines represent the toe off event. Horizontal lines represent the non-affected right side (blue), the affected left side (red), and reference data (green). Flexion is positive, and extension is negative. The gait curves at four time points illustrate changes in the affected side recorded before and after the intervention. Hip flexion and knee flexion of the affected leg increased during the swing phase, especially at the 3-month follow-up.

Individual augmented feedback was given to each participant at the follow-ups based on information from the gait reports from four test occasions (pre, post, 3-month and 6-month follow-ups). The KR comprised quantitative data from the gait reports and clinical outcome measures of the 10MWT and the 6MWT. The KP comprised qualitative data based on pre-selected gait curves for the 10MWT (Figure 2). Kinematic data in the sagittal plane for the lower limbs were chosen for the feedback situation since they are considered highly reliable in individuals post-stroke [25]. An additional rationale for selecting gait curves in the sagittal plane is their ease of explanation in terms of a gait cycle and its various phases. A positive shift in the curves was observed when the sagittal plane motion became more similar to the reference data, indicating the reappearance of premorbid gait patterns. Conversely, a negative shift was noted when the sagittal plane motion became less similar to the reference data, suggesting the emergence of compensatory adjustments and changes in the gait pattern. In the feedback scenario, the physiotherapists highlighted the positive shifts in outcome measures that they deemed crucial to walking ability. Thereafter, the participant was instructed to regularly perform specific home exercises based on the results of the gait reports.

### 2.5. Data Analysis

The spatiotemporal variables included time, cadence, and speed. The kinematic variables were sagittal range of motion (ROM), peak flexion, and peak extension in the hip and knee joints. All variables are considered reliable for assessing gait post-stroke [25]. To evaluate the 6MWT, we analysed data from three 25-metre intervals that were defined as tracks according to Pollet et al. [26]: the first track completed at the beginning (T0), the track completed at the third minute of the test (T1), and the last completed track before the end of the test (T2).

Gait parameters, due to sample size, were analysed based on the minimal detectable change (MDC) for chronic stroke [25,27,28]. This represents the amount of change for a single individual that must be observed before a change can be considered to exceed the measurement error and variability, with a 95% confidence interval (MDC_95_).

## 3. Results

All participants completed the group intervention within the clinic, received instructions for exercises to be performed at home, and completed the 3-month and 6-month follow-up sessions. Each participant attended every session during the intervention and every follow-up in the same group. No adverse events were reported.

### 3.1. Outcome Measures of the 10MWT Comfortable Speed

Participants A, B, and C demonstrated the most evident improvements (changes ≥ MDC) from pre-intervention to the 3-month follow-up (Table 2 and Table 3). Participant A increased their speed by 0.18 m/s, cadence by 10 steps, hip flexion by 10.3°, and hip ROM by 6.2°. Participant A also increased knee flexion by 28.1° and knee ROM by 29.1°, improvements that somewhat decreased at the 6-month follow-up. Participant B increased their speed by 0.15 m/s, an improvement that increased to 0.18 m/s after 6 months. Participant B also increased knee flexion by 8.5° and knee ROM by 13.3°. Participant C increased their speed by 0.22 m/s, an improvement that decreased to 0.18 m/s at the 6-month follow-up. Participant C also increased hip flexion by 10.9° and hip ROM by 13.2°.

### 3.2. Outcome Measures of the 10MWT Fast Speed

Participants A, B, and C showed the most evident improvements from the pre-intervention to the 3-month follow-up (Table 2 and Table 4). Participant A increased their hip flexion by 10.8°, hip extension by 8.4°, and knee ROM by 15.1°. Participant A also increased their knee flexion by 14.6°, an improvement that increased to 19.5° at the 6-month follow-up. Participant B increased their hip ROM by 9.9°, knee flexion by 8.2°, and knee ROM by 13°. Participant B also increased their hip extension by 11.6° after 6 months. Participant C increased their speed by 0.25 m/s and increased their hip flexion by 16.5° at the 3-month follow-up, an improvement that decreased to 10.9° at the 6-month follow-up. Participant C also increased their hip ROM by 8.6° after at the 3-month follow-up and their knee flexion by 17.8° at the 6-month follow-up.

### 3.3. Outcome Measures of the 6MWT

**Figure 3:**
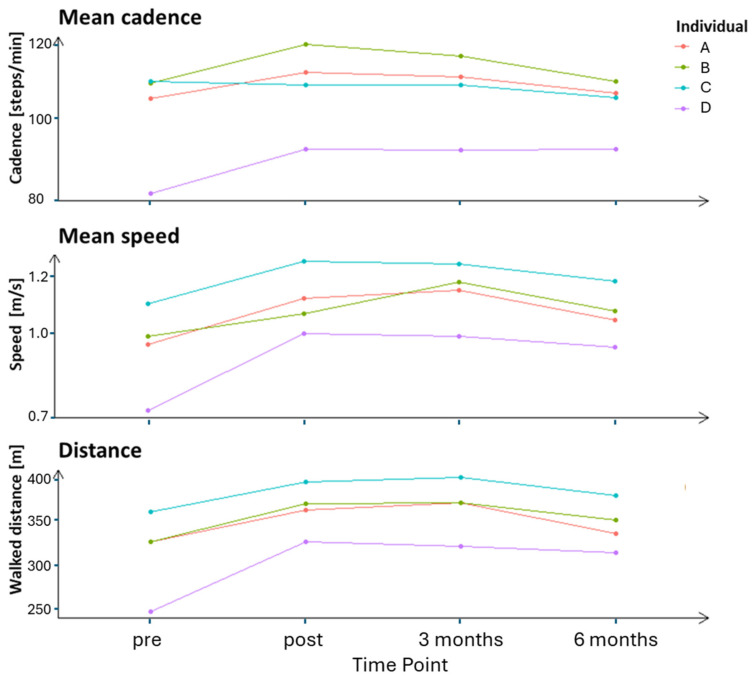
Outcome measures of the 6MWT across four measurement time points for all four tested individuals.

Participants A and B increased their walking distance by 45 m and mean speed by 0.20 m/s at the 3-month follow-up, improvements that declined at the 6-month follow-up (Figure 3). Participant D increased their walking distance by 80 m, mean cadence by 11.3 steps/min, and mean speed by 0.27 m/s at the post-intervention, improvements that were retained over time to the 6-month follow-up. Analysed tracks of the 6MWT showed that Participant D increased speed all through the 6MWT (T0-T2) from the post-intervention to the 6-month follow-up (Table 5). Participants A and B increased their speed during the first two tracks of the test (T0–T1), while Participant C increased speed during the last track of the test (T2) at the 3-month follow-up.

### 3.4. Patient-Reported Questionnaire

Each participant indicated a keen interest in monitoring the progression of gait curves over time and comparing their findings with reference data (Table 6). They rated their understanding of the gait curves as *good* to *very good*. Also, they scored their understanding of movement problems higher than their understanding of potential compensatory movements underlying their gait patterns. Participant A commented that it was easy to see the changes in the gait curves, although the gait reports contained a lot of information. Notably, Participant A perceived no compensatory movements and scored zero on that question. Participant B thought it was interesting to see proof of improvements. Likewise, Participant C thought it was interesting to see what makes a difference and what needs to be trained. Participant D thought the knee curves were the easiest to understand.

## 4. Discussion

This study details the results associated with the addition of augmented feedback in LE-CIMT derived from sensor-based gait reports in four participants with chronic stroke. Employing established MDCs for various gait parameters and comparing participants’ gait data with reference data allowed for a more nuanced understanding and interpretation of individual changes in walking ability and whether changes could be considered improvements or deteriorations. Improvements in gait parameters (such as increased walking distance, gait speed, joint angles, and cadence) changed over time and were most obvious at the 3-month follow-up. These improvements showed that the changes in gait patterns became more similar to the reference data, indicating the reappearance of normal gait patterns (recovery), whereas some changes in gait patterns became more distinct from the reference data, indicating adjustments of gait patterns (compensation). In addition, some changes in the gait patterns seemed to result from a combination of both recovery and compensatory movements. All participants rated a high interest in receiving information derived from the gait reports. Also, they found that the gait curves were understandable when feedback was given about their gait patterns. However, comments from one participant indicated some difficulty in understanding whether their walking ability was affected by stroke-related movement problems or by compensatory movement strategies.

Regarding the 10MWT, the results of our small clinical sample were in line with the results of a recent study that investigated 147 middle-aged patients with stroke who performed modified LE-CIMT by using a comparable protocol [20]. In that study, Marklund et al. reported that the patients demonstrated improved walking ability in terms of speed and distance directly after the intervention and 3 months later. However, that study did not include a 6-month follow-up or an evaluation of kinematic parameters. While Participants A, B, and C showed improvement in certain gait parameters of the 10MWT (changes ≥ MDC), there were also observed contradictory changes (i.e., appearance of alternative gait patterns). For instance, Participants A and B exhibited improved knee flexion during the swing phase after 3 months but concurrently experienced excessive knee hyperextension during the stance phase. This result prompted the decision to introduce additional home exercises with knee control, including crouch gait. Participants A and B subsequently exhibited a slight decrease in knee hyperextension after 6 months, although this was not different in comparison to the baseline assessments. Participant C demonstrated improved knee flexion during the swing phase at the post-intervention but simultaneously demonstrated excessive knee flexion during the stance phase. This contradictory result prompted the participant’s decision to refrain from home exercises involving a crouch gait. Accordingly, Participant C exhibited reduced knee flexion during the stance phase after 6 months (comfortable speed), although this was not different in comparison to the baseline assessments. For Participant D, there were no discernible improvements in the spatiotemporal and kinematic parameters of the 10MWT after the intervention. The absence of improvements (changes < MDC) for Participant D was probably due to the sensory loss of the affected knee at baseline that was identified distally and thereby made the incorporation of external feedback and motor learning more difficult. When sensory information is not effectively integrated by the CNS following a stroke, interventions aimed at restoring a more symmetrical gait may prove ineffective [29].

Regarding the 6MWT, Participants A, B, and D improved their walking distance, particularly after 3 months. Participant C also increased their distance, but not at the level of the MDC. The discrepancy in changes in gait speed during the execution of tracks among our participants aligned with earlier studies of persons post-stroke [26,30,31]. Different strategies (e.g., start at a high speed and decline over time or start at a lower speed and maintain the speed) may contribute to gait changes during the 6MWT [26]. In contrast to the results of the 10MWT, Participant D showed improvement in the spatiotemporal outcomes of the 6MWT directly after the intervention, an improvement that slightly declined at the follow-ups. These improvements were probably due to increased endurance and higher certainty of their ability to walk longer distances without aids. This assumption is consistent with previous research that has shown that LE-CIMT improves outcome measures such as oxygen uptake and balance [13].

The LE-CIMT protocol has evolved since the inception of our study, and it continues to undergo further development [32,33,34,35]. A pilot study evaluating the effects of an extended LE-CIMT programme (an intense 3-week-long initiation phase and a less-intense 6-month-long maintenance phase, including conventional physiotherapy) found significant improvements in the 10MWT (speed and cadence) and the 6MWT (distance) [35]. Previous studies on the effects of the LE-CIMT have used optoelectronic systems [36], instrumented walkway systems [37], and computerised force platform systems [38]. Although portable systems have been suggested to be useful for quantifying movements and measuring objective effects of the CIMT [39], there is no previous study that has included augmented feedback derived from sensor-based gait reports. In a recent study, sensor-derived movement reports were used to provide terminal visual feedback (KR) to stroke survivors in a natural environment over a one-week monitoring period [40]. The findings of that study emphasised the continued use of sensor-based feedback as a complement to clinical stroke care and to motivate engagement outside clinical settings. The authors also discussed the challenges related to providing sensor-based data that are used by both stroke survivors and clinicians. They felt it was a delicate balance between ease of understanding for stroke survivors and sufficiently informative data to guide individualised treatment [40]. This was something we also experienced. Another challenge when using wearable technology may be to select key parameters among the various of gait features that can be measured [41]. Regarding gait assessments after stroke, gait speed and cadence are the most widely used outcome measures [42], while angular changes are rarely assessed [43]. Our study stands out for its innovative use of wearable IMU sensors to measure both spatiotemporal and sagittal joint angles as outcome measures when assessing the effects of LE-CIMT on post-stroke gait. Additionally, by incorporating augmented feedback in a real-world clinical setting, it offers valuable insights for developing personalised rehabilitation programmes.

### Methodological Considerations

A strength of this study is its longitudinal design with a predefined protocol and the inclusion of the participants’ perspectives. This study was conducted in a clinical context with available clinical resources. Each test was only performed once and conducted with a standing start in accordance with existing clinical routines. To enhance the reliability of our gait data, the first and last gait cycles of each trial were excluded from the analysis to eliminate the acceleration and deceleration phases. The limited length of the available corridor at the clinic restricted the walking course of the 6MWT. Consequently, our participants walked between two cones that were 25 m apart instead of the recommended 30-metre walking course [44]. Despite our test procedure being somewhat different than those of the selected studies that reported the applied MDC values [25,27,28], we considered these values appropriate to use for the post hoc analysis. However, the generalisability of this study should be interpreted with caution due to the small sample size and lack of a control group. Another concern was that the augmented feedback from the sensor-based gait reports was only provided during the follow-up assessments. We plan to address identified limitations of the case series in a future larger cohort. The self-reported questionnaire can be improved by asking the two last questions differently or as a single question. Further, walking at a self-selected speed for 2 min on a 14-metre walking path with cones at both ends might be a more feasible gait test in clinical settings [45]. Also, reducing the number of sensors may be relevant to achieve a more useful IMU solution for both clinical and home assessment [41,46].

## 5. Conclusions

The integration of sensors significantly enhances traditional clinical assessments by providing deeper insights into gait dynamics. This empowers individuals post-stroke to optimise their treatment outcomes through informed feedback and personalised interventions. To validate these findings and further explore the clinical utility of wearable IMU sensors in stroke rehabilitation, larger randomised controlled trials are necessary. Additionally, developing real-time feedback applications could enhance clinical assessments, improve patient engagement, and enable personalised treatment plans in a clinical setting. Increasing sample sizes in these studies would provide more robust data.

## Figures and Tables

**Table 1:** Characteristics of the participants.

	A	B	C	D
Age (years)	25	57	55	56
Sex (female/male)	F	F	M	M
Affected side (right/left)	L	R	R	R
Time since stroke (months)	14	10	15	12
Type of stroke (ischemic/haemorrhagic)	H	H	I	H
Body Mass Index (kg/m^2^)	19.6	23.7	24.6	27.5
FMA-LE (scores)				
Total motor function (maximum 34)	27	25	23	26
Sensation (maximum 12)	9	12	10	6
Passive joint motion (maximum 20)	20	20	20	20
Pain (maximum 20)	20	20	20	20
Walking aids	No	No	No	Yes

FMA-LE = Fugl–Meyer Assessment Lower Extremity.

**Table 2:** Spatiotemporal variables of the 10MWT.

		10MWT—Comfortable Speed			10MWT—Fast Speed		
Participants	Occasion	Time (s)	Cadence (steps/m)	Speed (m/s)	Time (s)	Cadence (steps/m)	Speed (m/s)
A	Pre	14.05	94.05	0.71	10.04	118.23	1.00
	Post	12.48	95.15	0.80	9.15	121.9	1.09
	3 months	11.24	**104.13**	**0.89**	8.92	117.75	1.12
	6 months	13.12	96.42	0.76	9.59	118.28	1.04
B	Pre	13.18	103.40	0.76	8.94	134.25	1.12
	Post	12.30	109.11	0.81	8.04	**142.91**	1.24
	3 months	10.97	103.69	**0.91**	7.90	129.51	1.27
	6 months	10.59	110.33	**0.94**	7.69	130.27	1.30
C	Pre	13.82	95.67	0.72	9.10	108.38	1.10
	Post	11.82	99.34	0.85	8.76	109.46	1.14
	3 months	10.61	100.72	**0.94**	7.42	112.24	**1.35**
	6 months	11.10	100.93	**0.90**	7.94	116.61	1.26
D	Pre	22.04	67.21	0.45	10.18	106.52	0.98
	Post	20.65	68.73	0.48	9.24	102.22	1.08
	3 months	19.97	67.81	0.50	9.55	108.46	1.05
	6 months	20.79	66.07	0.48	10.38	100.85	0.96

10MWT = Ten-Metre Walk Test; Pre = pre-intervention (i.e., the day before the start of the intervention); Post = post-intervention (i.e., the day after the end of the intervention). Bold values indicate improvements according to the estimated minimal detectable change (MDC) (cadence = MDC95 ≥ 8.58 steps/min, comfortable speed = MDC95 ≥ 0.15 m/s and fast speed = MDC95 ≥ 0.19 m/s).

**Table 3:** Kinematic variables of the 10MWT—Comfortable speed.

		Hip Joint			Knee Joint		
Participants	Occasion	Max Angle °	Min Angle °	ROM °	Max Angle °	Min Angle °	ROM °
Reference data		36.07 (4.46)	−15.35 (3.80)	51.42 (4.69)	61.55 (7.47)	2.45 (4.06)	59.09 (6.64)
A	Pre	19.94	−28.40	48.34	25.09	−12.85	37.94
	Post	24.65	−21.41	46.06	**37.71**	−13.82	**51.53**
	3 months	**30.23**	−24.33	**54.56**	**53.16**	−13.87	**67.03**
	6 months	21.89	−22.94	44.82	**46.07**	−10.06	**56.13**
B	Pre	34.62	−13.79	48.40	39.74	−9.79	49.52
	Post	32.74	−15.70	48.45	43.09	−9.56	52.65
	3 months	38.83	−19.37	58.20	**48.27**	−14.52	**62.79**
	6 months	33.86	−19.51	53.36	43.93	−11.40	55.32
C	Pre	27.75	−7.61	35.36	41.78	7.48	34.31
	Post	31.88	−2.93	34.81	**59.43**	24.54	34.89
	3 months	**38.61**	−9.89	**48.51**	45.94	17.35	28.59
	6 months	26.79	−10.47	37.26	39.99	10.81	29.18
D	Pre	22.33	−18.44	40.76	12.96	−0.77	13.73
	Post	19.51	−19.86	39.37	4.71	−7.59	12.30
	3 months	28.90	−12.66	41.56	14.82	0.89	13.93
	6 months	19.09	−23.26	42.35	10.85	−6.14	16.98

10MWT = Ten-Metre Walk Test, MDC = minimal detectable change; Pre = pre-intervention (i.e., the day before the start of the intervention); Post = post-intervention (i.e., the day after the end of the intervention). Bold values indicate improvements according to the minimal detectable change (MDC) estimates (hip peak flexion = MDC95 ≥ 9.01°, hip peak extension = MDC95 ≥ 7.56°, hip ROM = MDC95 ≥ 4.69°, knee peak flexion = MDC95 ≥ 6.54°, knee peak extension = MDC95 ≥ 5.90°, and knee ROM = MDC95 ≥ 6.43) and in relation to reference data.

**Table 4:** Kinematic variables of the 10MWT—Fast speed.

		Hip Joint			Knee Joint		
Participants	Occasion	Max Angle °	Min Angle °	ROM °	Max Angle °	Min Angle °	ROM °
Reference data		42.94 (5.86)	−17.93 (4.41)	60.87 (5.41)	63.99 (8.73)	0.65 (5.11)	63.34 (8.18)
A	Pre	24.41	−27.12	51.53	38.42	−10.26	48.68
	Post	26.44	−21.94	48.38	41.87	−17.47	59.34
	3 months	**35.17**	**−18.75**	53.92	**52.98**	−10.78	**63.76**
	6 months	23.48	−27.07	50.55	**57.94**	−13.73	71.67
B	Pre	45.20	−6.30	51.49	45.85	−0.24	46.09
	Post	54.25	−0.47	54.72	50.36	−2.29	52.65
	3 months	50.25	−11.14	**61.40**	**54.03**	−5.10	**59.13**
	6 months	36.68	**−17.89**	54.57	48.09	−8.75	56.83
C	Pre	27.36	−21.50	48.86	28.05	−1.97	30.02
	Post	35.05	−8.43	43.48	**44.74**	15.81	28.93
	3 months	**43.87**	−13.60	57.47	34.42	3.50	30.92
	6 months	**38.25**	−10.51	48.76	**45.83**	18.99	26.84
D	Pre	37.52	−16.28	53.80	28.05	4.98	23.07
	Post	29.18	−26.32	55.50	17.25	−4.02	21.27
	3 months	14.82	0.89	13.93	23.52	5.33	18.20
	6 months	10.85	−6.14	16.98	21.62	−1.01	22.62

10MWT = Ten-Metre Walk Test, MDC = minimal detectable change; Pre = pre-intervention (i.e., the day before the start of the intervention); Post = post-intervention (i.e., the day after the end of the intervention). Bold values indicate improvements according to the minimal detectable change (MDC) estimates (peak hip flexion = MDC95 ≥ 9.01°, peak hip extension = MDC95 ≥ 7.56°, hip ROM = MDC95 ≥ 4.69°, peak knee flexion = MDC95 ≥ 6.54°, peak knee extension = MDC95 ≥ 5.90°, and knee ROM = MDC95 ≥ 6.43) and in relation to reference data.

**Table 5:** Spatiotemporal variables of the 6MWT.

Participant	Variable	Occasion	T0	T1	T2
A	Cadence	Pre	108.55	104.93	102.41
	(steps/min)	Post	114.26	113.40	108.67
		3 months	112.05	110.13	108.67
		6 months	109.74	105.95	104.25
	Speed	Pre	0.94	0.97	0.95
	(m/s)	Post	1.10	1.19	1.06
		3 months	**1.17**	**1.19**	1.08
		6 months	1.08	1.04	1.00
B	Cadence	Pre	109.8	109.59	108.67
	(steps/min)	Post	**125.28**	115.87	116.41
		3 months	**122.89**	113.89	110.13
		6 months	112.05	108.70	108.24
	Speed	Pre	0.95	0.96	1.04
	(m/s)	Post	1.01	1.12	1.05
		3 months	**1.25**	**1.17**	1.11
		6 months	1.05	1.06	1.11
C	Cadence	Pre	109.26	109.27	110.52
	(steps/min)	Post	112.04	104.32	109.99
		3 months	110.38	106.80	109.28
		6 months	106.23	106.22	104.63
	Speed	Pre	1.14	1.08	1.08
	(m/s)	Post	1.27	1.24	**1.25**
		3 months	1.22	1.20	**1.30**
		6 months	1.14	1.21	1.19
D	Cadence	Pre	79.29	81.55	82.32
	(steps/min)	Post	**97.61**	88.24	**112.40**
		3 months	**98.63**	**91.97**	90.82
		6 months	93.61	**93.79**	89.60
	Speed	Pre	0.70	0.72	0.73
	(m/s)	Post	**1.01**	**0.97**	**0.98**
		3 months	**1.00**	**1.00**	**0.95**
		6 months	**0.94**	**0.94**	**0.95**

6MWT = Six-Minute Walk Test; Pre = pre-intervention (i.e., the day before the start of the intervention); Post = post-intervention (i.e., the day after the end of the intervention). T0 = the first 25-metre interval (track) completed at the beginning, T1 = the track completed at the third minute of the test, and T2 = the last completed track before the end of the test. Bold values indicate improvements according to the published minimal detectable change (MDC) (cadence = MDC95 ≥ 8.58 steps/min, speed = MDC95 ≥ 0.17 m/s).

**Table 6:** Results of participant-reported questionnaire using a 10-point scale.

Perception Aspects	A	B	C	D
Understanding of gait curves ^a^	5	8	8	9
Interest in gait curves ^b^	10	10	10	10
Comparison of own results with reference group’s curves ^b^	10	10	10	8
Following development over time ^b^	10	10	10	10
Understanding of movement problems ^c^	10	10	10	9
Understanding of compensatory movements ^c^	0	6	9	6

^a^ 0 = very bad, 10 = very good; ^b^ 0 = not at all interested, 10 = very interesting; ^c^ 0 = do not agree at all, 10 = completely agree.

## Data Availability

The data presented in this study are available upon request from the corresponding author. The data are not publicly available due to a lack of consent for sharing individual data.
